# Andreev Molecule in Parallel InAs Nanowires

**DOI:** 10.1021/acs.nanolett.1c01956

**Published:** 2021-09-20

**Authors:** Olivér Kürtössy, Zoltán Scherübl, Gergö Fülöp, István Endre Lukács, Thomas Kanne, Jesper Nygård, Péter Makk, Szabolcs Csonka

**Affiliations:** † Department of Physics and Nanoelectronics “Momentum” Research Group of the Hungarian Academy of Sciences, Budapest University of Technology and Economics, Budafoki út 8, 1111 Budapest, Hungary; ‡ University of Grenoble Alpes, CEA, Grenoble INP, IRIG, PHELIQS, 38000 Grenoble, France; § Center for Energy Research, Institute of Technical Physics and Material Science, Konkoly-Thege Miklós út 29-33, H-1121, Budapest, Hungary; ∥ Center for Quantum Devices, Niels Bohr Institute, University of Copenhagen, 2100 Copenhagen, Denmark

**Keywords:** Andreev molecule, Yu-Shiba-Rusinov, superconductivity, nanowire, hybridization

## Abstract

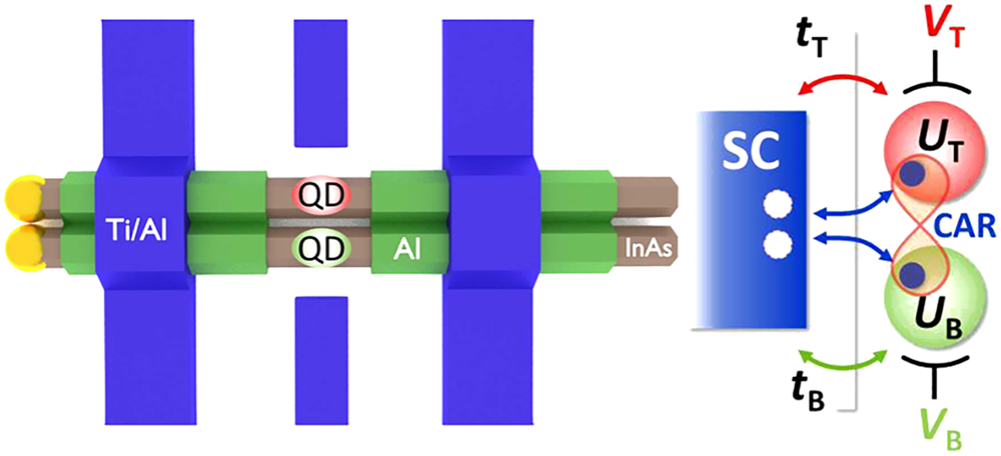

Coupling individual atoms fundamentally changes the state of matter: electrons bound to atomic cores become delocalized turning an insulating state to a metallic one. A chain of atoms could lead to more exotic states if the tunneling takes place via the superconducting vacuum and can induce topologically protected excitations like Majorana or parafermions. Although coupling a single atom to a superconductor is well studied, the hybridization of two sites with individual tunability was not reported yet. The peculiar vacuum of the Bardeen–Cooper–Schrieffer (BCS) condensate opens the way to annihilate or generate two electrons from the bulk resulting in a so-called Andreev molecular state. By employing parallel nanowires with an Al shell, two artificial atoms were created at a minimal distance with an epitaxial superconducting link between. Hybridization via the BCS vacuum was observed and the spectrum of an Andreev molecule as a function of level positions was explored for the first time.

## Introduction

**Figure 1:**
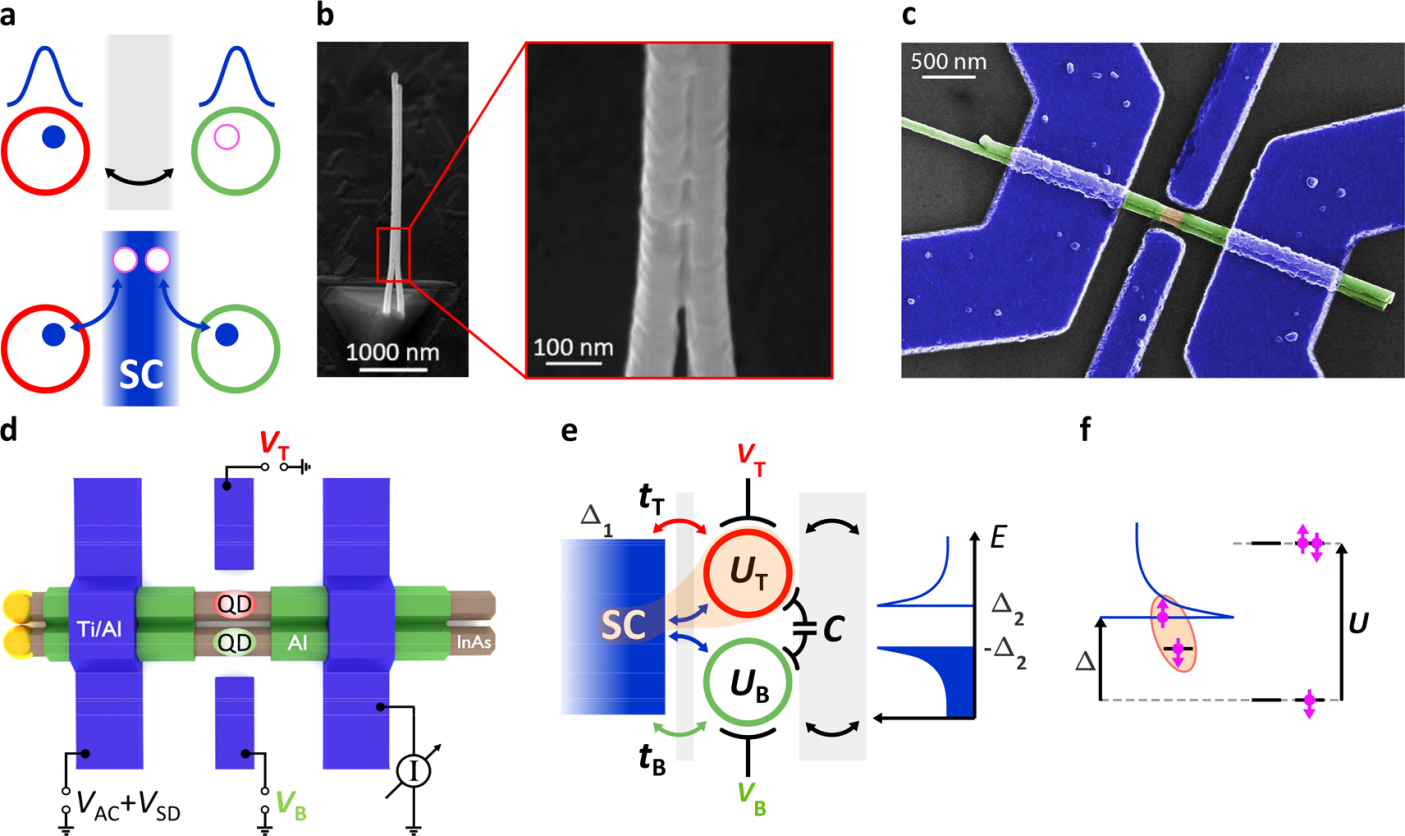
(a) General concept of a molecular state formed by tunneling via a barrier between two atomic sites (top). In our case, the interaction between the QDs is mediated by an SC (bottom), where two electrons can be created from the SC vacuum. (b) High-resolution scanning electron micrograph (SEM) of the as-grown parallel wires. The epitaxial Al connects the two InAs nanowires. (c) False-color SEM and (d) schematic illustration of the device. The epitaxial Al shell (green) is etched in the middle where the QDs are formed in the two InAs wires (brown). The QDs are tuned by the two side gates with gate voltages *V*_T_ and *V*_B_, while the differential conductance *G* = *I*_AC_/*V*_AC_ is measured in two-terminal measurements between evaporated Ti/Al (blue) contacts. (e) Sketch of the setup used for modeling the system with tunnel coupling *t*_T_ (*t*_B_), charging energy *U*_T_ (*U*_B_), and on-site energy ε_T_ (ε_B_) controlled by *V*_T_ (*V*_B_) belonging to the top (bottom) QD. Gray rectangles illustrate the tunnel barriers. While the left electrode with a gap of Δ_1_ was strongly coupled to the QDs, the right one was weakly coupled, thus the latter served as a BCS probe with a gap of Δ_2_. Interdot capacitance *C* was also considered. (f) Formation of YSR singlets in a SC-QD system (marked by orange in panel e). In the *U* > Δ limit, a quasi-particle and an electron on the QD create a bound state inside the gap.

On the basis of Bardeen–Cooper–Schrieffer (BCS) mean-field theory,^1^ the superconducting vacuum only allows the addition of individual electrons with energy above the superconducting gap, however, it serves as a free source and drain of electron pairs, known as Cooper pairs (see Figure 1a). The interplay between an artificial atom, namely a quantum dot (QD), and the BCS vacuum was studied intensively, contributing to the formation of a subgap excitation, a so-called Yu–Shiba–Rusinov (YSR) state (or called Andreev Bound states in other limits, see Figure 1f).^2−14^ These excitations are shared between the QD and the superconductor (SC).^13^ Two of such bound states, which are formed with two spatially separated QDs, could be hybridized by the common SC lead, which we call an Andreev molecule.^15^ Coupling two QDs to a joint SC is also a basic building block of a Cooper pair splitter (CPS),^16^ where the QDs are attached to two normal leads allowing one to create spatially separated entangled electron pairs^17−22^ via crossed Andreev reflection.^23−25^ While a CPS favors weak SC-QD couplings, an Andreev molecule requires the opposite limit. Several theoretical works investigated how two bound states localized on separated dots effectively couple via the SC,^15,26−35^ for example, as a minimal model for Majorana chain.^36^ Hybridization of YSR states was studied recently in a few scanning tunneling microscopy measurements placing different dimers on an SC surface.^37−41^ The target of the present work is to realize an Andreev molecular state with artificial atoms allowing individual tunability of the atomic sites and to explore its spectrum^15^ as a function of level position for the first time.

Realization of an Andreev molecule with QDs imposes a set of challenging constraints: the QDs must be strongly coupled to the SC and their distance should be minimized while preventing direct tunneling between them.^42,43^ To fulfill these requirements we construct our artificial atoms in a novel superconducting hybrid nanostructure, where double InAs nanowires are grown in close vicinity and are connected by an epitaxial SC Al shell (see Figure 1b).^44−46^ Whereas two QDs can be formed in separate wires thereby excluding the direct tunneling between them, the epitaxial Al shell yields a defect-free SC–semiconductor interface ensuring the strong proximity^47,48^ and SC-QD coupling as used in various hybrid quantum devices, like Andreev-qubits,^49−52^ Gatemons,^53,54^ or Majorana devices.^55−61^ Recent works have already reported the Cooper pair splitting signals^62^ and nonlocal pair tunnelings^63^ in individual nanowires placed parallel close to each other manually with a micromanipulator. Moreover, Andreev bound states were also coupled by direct tunneling between QDs in series.^64^ However, none of them has realized the strong hybridization of artificial atoms via a SC needed for the formation of the Andreev molecular state, the elementary building block of a Majorana chain.^36^ In this paper, we report the signature of an Andreev molecule, for the first time in parallel InAs nanowires. We discuss first the case of uncoupled YSR states and then compare it to the strongly interacting system involving the hybridization via SC and Coulomb repulsion, both experimentally and theoretically.

## Results and Discussion

### Device Outline

The specific system studied here is illustrated in Figure 1c–e. A parallel double QD was formed in a pair of InAs nanowires merged by epitaxial full-shell Al,^44^ which was etched away on a ∼250 nm long segment. Two common superconducting (Ti/Al) electrodes were attached to epitaxial Al on the nanowires forming parallel SC–QD–SC junctions in the two wires. Low-temperature electronic transport measurements were carried out at a base temperature of 40 mK (for details, see Methods). In two-terminal subgap spectroscopy, the differential conductance *G* = *I*_AC_/*V*_AC_ was measured with the tuning of the QDs by individual plunger gates, as depicted in Figure 1d (*V*_T_ corresponds to the top, *V*_B_ to the bottom gate voltage). The source terminal biased with *V*_SD_ was found to be coupled strongly to the QDs, whereas the other one worked as a SC tunnel probe leading to a SC–QD–I–SC junction, where I stands for insulator. Two different devices are presented in this paper; one did not show strong coupling between nanowires and thus serves as a reference junction (device A), whereas for the other a strong hybridization of the QDs and signatures of the Andreev molecular states was observed (device B).

**Figure 2:**
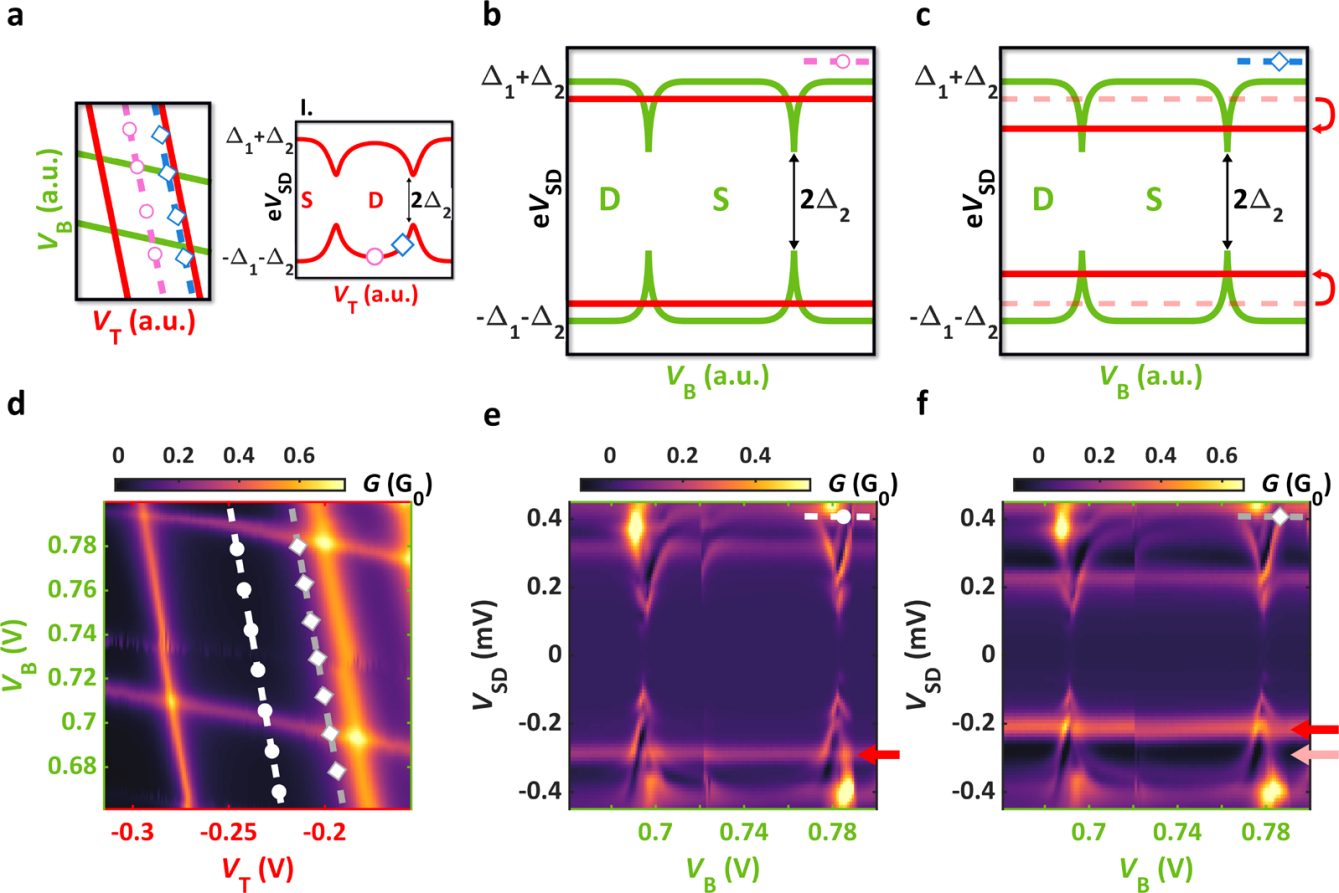
Uncoupled parallel YSR states (device A). (a) Schematic illustration about the stability map of parallel QDs with joint electrodes. Resonances of the top and bottom QDs are depicted with red and green, respectively. The lever arms refer to a finite cross capacitance of each gate to the opposite QDs. Inset I depicts the YSR spectrum residing in the top QD (“S” and “D” refer to singlet and doublet ground states, respectively). Δ_1_ and Δ_2_ are the superconducting gaps of the strongly coupled electrode and the SC probe. The pink circle and the blue diamond indicate the energy of YSR_T_ along the cuts taken in the stability map. (b) Expected excitation spectrum of YSR states along the pink line shown in panel a. Whereas YSR_B_ (green) evolves along the cut since it is sensitive to its own gate (*V*_B_), the YSR_T_ state (red) stays on constant energy as the slice is parallel to the red resonances. (c) Bound state spectrum along the blue line in panel a. The excitation of the YSR_T_ state moved to lower energy compared to the one in panel b (the original energy is depicted with pink dashed lines) as the charge degeneracy of the top QD was approached (see the blue diamond in inset I of panel a). (d) Measured conductance as a function of gate voltages for device A in the normal state. (e) Finite-bias spectroscopy measurement along the white dashed line depicted in panel d (superconducting state). Panel b illustrates well the experimental findings. The red arrows mark the excitations of YSR_T_. (f) Finite-bias spectroscopy measurement along the gray dashed line with a diamond in panel d, closer to the resonance of the top QD matching to panel c. The pink arrow indicates the position of the YSR_T_ signals in panel e.

When a QD is coupled strongly to an SC electrode, subgap states are formed by the hybridization between a QD level and the SC electrode.^2−4,12^ Let us consider the QD being singly occupied (called doublet ground state due to degeneracy in the spin of freedom, noted by “D”). In this case, the lowest excitation available is either adding an electron costing the charging energy, *U*, or adding a quasi-particle to the SC band, which costs the energy of the superconducting gap, Δ. However, the interaction between the QD and the SC induces (see Figure 1f) a lower-lying excitation: a singlet (marked by “S”) formed by an electron on the QD and a quasi-particle in the SC is hybridized with the empty QD and quasi-particle state via a Cooper pair transfer to the superconducting condensate. This results in a so-called YSR subgap state in the *U* > Δ limit (orange state in Figure 1f). In a QD, the level position can be tuned, and the YSR energy develops in an “eye-shaped” curve as a function of it,^11,12^ that is, the plunger gate voltage similarly to the one sketched in inset I. of Figure 2a. For large (or small) enough gate voltage, the ground state of the QD changes to the singlet state bearing double (or zero) occupation (for more details see Supporting Information or, for example, ref (14)).

In the following, we review the spectrum of the parallel double QD structure in three steps: (i) two independent, uncoupled YSR states, (ii) adding interdot Coulomb repulsion, and (iii) including the superconducting coupling. We label the QDs and their features as top (T) and bottom (B) ones, marked with red and green as in Figure 1, respectively, supposing only a single YSR state residing in each QD (YSR_T_ and YSR_B_).

### Uncoupled YSR States

The parallel YSR states in uncoupled wires are discussed in Figure 2 (top row expectations, bottom row measurements). Figure 2a illustrates the zero-bias conductance of the two wires as a function of the two plunger gate voltages in the normal state. Here the interdot capacitance is negligible, however, there is a finite cross-capacitance between the top (bottom) plunger gate and the bottom (top) QD, resulting in the tilted lines in the phase diagram.^65^Figure 2b,c illustrates the finite-bias spectrum along the pink and blue dashed line in Figure 2a parallel to the top QD resonances as a function of *V*_B_. Asymmetric coupling of the wires, *t*_T_ > *t*_B_ and different superconducting gaps of Δ_1_ and Δ_2_ for the strongly coupled electrode and the SC probe are considered, respectively, to reproduce the experimentally observed features. The spectroscopy yields the sum of an “eye-shaped” excitation (green) typical for YSR systems and an excitation line at constant energy (red). The green YSR patterns belong to the bottom QD (YSR_B_), while the red ones can be identified as YSR_T_ since the on-site energy of the top QD is kept constant due to the parallel slicing in both panels. The excitations do not touch at zero *V*_SD_ but stay always at finite energy originating from the SC tunnel probe, which introduces *a* ± Δ_2_ gap in the excitation spectrum. These minima correspond to the ground state transitions of the QD addressed also in the figure. Depending on the position of the slice, the energy of the constant line can vary between Δ_2_ and Δ_1_ + Δ_2_. Obviously, the YSR_T_ excitation can occupy the lowest energy Δ_2_ when the corresponding (top) QD is close to resonance (Figure 2c, blue line in Figure 2a), while moving deeper in the blockade brings its energy toward the gap edge Δ_1_ + Δ_2_ regardless of the parity of the ground state. The movement of the signal while approaching a resonance is indicated with red arrows in Figure 2c. For clarity, inset I in Figure 2a depicts YSR_T_ as the function of its own plunger gate (*V*_T_), in which the markers assign the actual excitation energies considered in Figure 2b,c.

The measurements of device A follow well our basic predictions outlined above. The stability map in the normal state, which was recorded by applying 250 mT out-of-plane magnetic field, is shown in Figure 2d. Two different bias cuts parallel to the top QD resonance in Figure 2e,f reveal the movement of YSR_T_ (red arrow) while the development of YSR_B_ remains intact. By a careful inspection of the data, one can identify an additional excitation line at higher energy, which can be attributed to another orbital of the bottom QD or to a higher-lying transition. Recording the spectrum along the other gate direction *V*_T_ a similar behavior of the bound states was observed (for details see Supporting Information), where the movements of (multiple) YSR_B_ states are also trackable. On the basis of the two dominant lever arms in the stability sweep and the fact that different YSR states were captured by tuning either *V*_T_ or *V*_B_ confirmed the model of having YSR states in both QDs.

We note that the Kondo effect^8,66,67^ was suppressed in most of the gate settings in both device A and B since the Kondo temperature did not exceed the superconducting gap (*k*_B_*T*_K_ < Δ_1_). As the QDs enter a more open regime, Kondo correlations appear competing with the superconductivity, which reduces the visibility of the outlined YSR behaviors (for details see Supporting Information).

### Interdot Coulomb Repulsion

**Figure 3:**
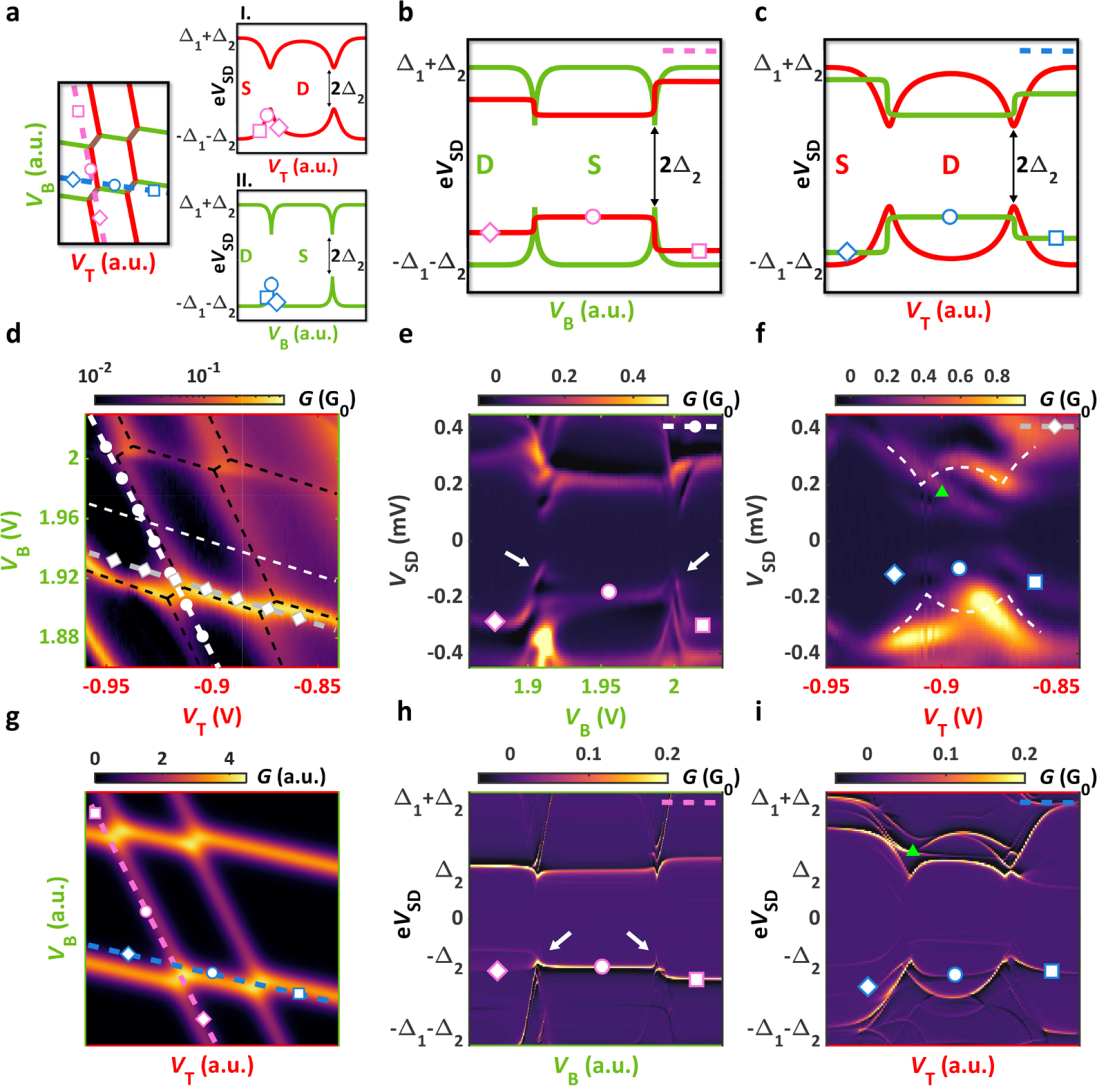
Coupled YSR states (device B). (a) Stability map of parallel QDs with strong interdot capacitance. Despite the pink and blue cuts being parallel to the resonances of the top and bottom QDs, respectively, the excitation energies of the neighboring YSR states shift while crossing the triple points. Markers in the insets indicate the neighboring YSR state energies along the cuts. (b) Predicted spectrum along the pink line (tuning the bottom QD) from panel a by considering the Coulomb interaction. In this case, steps in the energy of YSR_T_ are expected. (c) Similar spectrum to the one in panel b, but along the blue line in panel a resulting in the tuning of the top QD. (d) Normal state conductance as a function of the plunger gates on device B. Black dashed line illustrates the honeycomb structure attributed to the parallel QD system. (e) Bias spectroscopy measurement along the dashed line with circles depicted in panel d. Whereas most features show resemblance to panel b, anticrossings and bends toward zero energy occur at the charge degeneracy points marked by the white arrows. (f) Measured spectrum along the other dashed line with diamonds from panel d. The white dashed line shows the eye-shaped YSR_B_ doublet when it is recorded off-resonance with its trace depicted in panel d. Additional excitation lines, such as the one marked by the green triangle, also appear. In comparison to panel c, it can be seen that the measurements can not be described by the simple capacitive model. (g) Simulated normal state stability map reproducing the experimental data and panel a. (h) Numeric simulation of panel b with hybridization via the SC along the pink lines of panel g. The superconductivity induces bends, anticrossings, and conductance enhancement in YSR_B_. (i) Similar simulation but along the blue line in panel g. The enhanced YSR_B_ develops distinctly from the capacitive model and leads to distortions in YSR_T_. As one can see, the excitation lines are multiplied and not even symmetric in bias.

The question arises of how the spectrum is modified compared to the uncoupled case if there is significant interdot capacitance (see *C* in Figure 1e). The normal state stability map turns into the so-called honeycomb pattern (see Figure 3a) well-known for double QDs.^65^ Hence, slicing parallel to any resonances along a straight line in the gate map gives no longer a constant-energy YSR state but a charge state-dependent one. For example, along the pink dashed line, the top YSR state develops according to the symbols in inset I, where YSR_T_ is depicted as a function of its own plunger gate, *V*_T_. For small *V*_B_ values, the line cut is off-resonance and YSR_T_ is in the doublet ground state (diamond symbol). By increasing *V*_B_, the bottom QD is brought to resonance which leads to an effective gating of the top QD. This shifts the top QD closer to its resonance and lowers the energy of YSR_T_ (circle symbol). Going through another resonance of the bottom QD (by further increasing *V*_B_) displaces the top QD resonance again and its YSR state ends up in the singlet ground state (square symbol). These jumps of the signal at the charge degeneracy points, where the on-site energy of the QD changes abruptly, imply “steplike” excitations in total (see the red lines in Figure 3b). Analogously, a similar spectrum (shown in Figure 3c) is obtained along the blue line in Figure 3a with the symbols in inset II.

Whereas the uncoupled YSR states described well the behavior of device A, they clearly can not match the measurements on device B, shown in Figure 3d–f. Therefore, we now compare them to the simple case of having capacitive coupling between the two QDs. Figure 3d shows the measured normal state map providing qualitatively the same honeycomb structure (illustrated with black dashed lines) as the one in Figure 3a. Subgap spectroscopy was performed along the white and gray dashed lines. Similarly to device A, in the measurements of device B the conductance of YSR_T_ (red) was found to be larger than YSR_B_ (green) fulfilling the assumption of *t*_T_ > *t*_B_ already mentioned. In Figure 3e, the spectrum is presented as a function of *V*_B_ exhibiting similarities to Figure 3b. The YSR_B_ state is mostly bound to the gap edge and develops rapidly at the ground state transitions matching the green curve in Figure 3b. The YSR_T_ state (marked by the pink symbols at negative bias) also provides steplike features in accordance with the red curve in Figure 3b (marked by the pink symbols). Nonetheless, clear discrepancies emerge close to the charge degeneracy points (*V*_B_ = 1.91 V and *V*_B_ = 2 V). As the YSR_B_ and YSR_T_ excitations approach each other, they anticross, and YSR_T_ bends toward zero energy (indicated by white arrows) suggesting the hybridization of the states, which is unexpected in a simple capacitive picture. The difference between the predicted sketch and the measured data is more obvious for cuts along the other gate direction as the comparison of Figure 3c,f shows. Assuming only capacitive coupling between QDs (panel **c**), YSR_T_ is expected to take the red, eye-shaped curve as a function of *V*_T_. Such excitation is measured when YSR_B_ is far off-resonance (see the white dashed line in Figure 3f and its trace in Figure 3d, and also Supporting Information). However, the spectrum captured close to the resonance of YSR_B_ (along the gray dashed line in Figure 3d) strongly deviates from the expectation of simple capacitive coupling as the comparison of Figure 3f,c demonstrates. The unusual evolution of the signals was quite robust along any cuts taken in the vicinity of the charge degeneracies (for further data see Supporting Information).

### Superconducting Coupling

For the next step, we introduce a model, which goes beyond the interdot Coulomb interaction and takes into account the hybridization between the QDs via the SC. As shown below, our fully interacting two-dot simulation reproduces all of the main features of the unique experimental spectra.

To proceed, we modeled the QDs with single sites tunnel-coupled to the SC. The SC was treated in the zero bandwidth approximation^68,69^ by considering a single quasi-particle level, and the tunnel probe was handled perturbatively. In this subsystem, we calculated the eigenstates and the transport with exact diagonalization of the Hamiltonian (for detailed description of the model see Methods and Supporting Information). All relevant parameters (*U*_T_, *U*_B_, *C*, *t*_T_, *t*_B_, Δ_1_, Δ_2_, shown in Figure 1e) were directly extracted from the experimental data, thus the model has no fitting parameters. The simulated normal state stability map (Figure 3g) and the spectra taken along the pink and blue lines marked in Figure 3a,g are shown in Figure 3h,i.

After a quick comparison of Figure 3b,c with Figure 3h,i one can see that the hybridization via the SC strongly restructures the spectra. Let us now carefully compare the measurements in Figure 3e,f with the calculated spectra in Figure 3h,i and show that they qualitatively match well. (i) The anticrossings and the bends of YSR_T_ observed particularly in Figure 3e are restored in the numerical results (see white arrows in Figure 3h). As a result of using a superconducting tunnel probe, negative differential conductance around the YSR signals also appear in both of the experiments and the simulations.

In general, there are several characteristic features in Figure 3f, which strongly deviate from the capacitive picture, nevertheless, they are qualitatively recovered in the simulation in Figure 3i. First of all, (ii) the eye-shaped YSR_T_ resonance is completely distorted in the measurement as well as in the numerical data. Moreover, (iii) the expected horizontal YSR_B_ signal does not stay flat in the doublet region of YSR_T_ (indicated by the blue circle between *V*_T_ = −0.92 V and *V*_T_ = −0.87 V) but rather follows the curvature of YSR_T_ similarly to the simulation. Though well-pronounced anticrossings are absent, (iv) extra dispersive lines (one example is marked by the green triangle) arise between the YSR_T_ and YSR_B_ signals in, likewise in the theoretical curves. (v) It is also remarkable that the measured spectrum is asymmetric for the sign of the bias, which is also established in panel i. It is notable that (vi) the conductance of the YSR_B_, whose coupling is three times weaker than YSR_T_ in the model, is greatly enhanced and reaches ∼80% of the strongly coupled YSR_T_ near the charge degeneracy points, in accordance with the simulated spectra. Besides the good agreement in the listed properties (i–vi), the theory does not match the measurements in a few aspects. On one hand, the discrepancies originate from several simplifications the model takes. The calculations neglect the presence of multiple QD orbitals and exclude the relaxation from excited states, hence allowing arbitrary high-energy virtual states, which are usually not visible in a bias-spectroscopy measurement. On the other hand, further limitation comes from the broad line width of the measured YSR states smearing the neighboring excitation lines. Overall, despite the theory being simplified, many prominent features of the measured data were captured in the simulation qualitatively by assuming hybridization via the SC, which supports our interpretation of an observed Andreev molecule.

The two investigated devices (A and B) showed very different behaviors. Whereas for A no hybridization was observed, sample B exhibited signatures of the Andreev molecule. Careful SEM analysis revealed an important structural difference; for sample B the two InAs nanowires were merged by the epitaxial Al, whereas for device A the wires have separated and became only connected by the ex situ evaporated contacts (blue in Figure 1a, see Supporting Information). These Ti/Al contacts were established ∼400 nm away from the QDs, which could explain the absence of the SC-induced hybridization in device A.^16,70^

## Conclusions

In summary, we have found strong interactions between parallel YSR states realized in double InAs nanowires connected by an epitaxial Al shell. The small geometrical distance between the QDs resulted in capacitive coupling, while the shared epitaxial Al source contact enabled hybridization via the SC vacuum. The latter one allowed the emergence of an Andreev molecular state, whose spectrum was explored as a function of the QD level positions for the first time. The detected spectroscopic features were reproduced by our numerical calculations. Our result is an important milestone toward artificial topological superconducting systems, where Kitaev-like chains^31,55^ are assembled from sites hybridized via SCs.^36,64,71^ With the strong superconducting coupling demonstrated here, double InAs nanowires can be also promising candidates to host non-Abelian excitations, like parafermions^72^ as a key ingredient of topological quantum computation.^73,74^

## Methods

### Device Fabrication

In As nanowires were grown by MBE in the wurtzite phase along the ⟨0001⟩ direction catalyzed by Au. The pattern of the predefined Au droplets allowed one to control the geometrical properties of the proposed double nanowires, including the diameter, distance, and the corresponding alignment of the cross sections. The 20 nm thick full-shell Al was evaporated at low temperature in situ, by rotating the substrate, providing epitaxial, oxide-free layers. The evaporation on such a pair of adjacent nanowires resulted in the merging by the Al. Nanowires with ∼80 nm diameter and ∼4 μm length were deposited on a p-doped Si wafer capped with 290 nm thick SiO_2_ layer by using an optical transfer microscope with micromanipulators. The Al shell on a ∼ 250 nm long segment was removed by means of wet chemical etching. A MMA/MAA EL-6 double-layer performed as a masking layer, which was locally exposed by EBL, allowing the MF-321 selective developer to access the Al (60 s). The etching was followed by a careful localization of the wires with high-resolution SEM. Both source-drain and side gate electrodes were installed in a common EBL step. The sample was exposed to RF Ar milling in the evaporator chamber to remove the native Al_2_O_3_. The process was followed by the metallization of Ti/Al (5/95 nm) without breaking the vacuum.

### Experiments

Low-temperature characterization was carried out in a Leiden Cryogenics dry dilution refrigerator with a base temperature of 40 mK. Transport measurements were performed with a standard lock-in technique by applying 10 μV AC signal at 113 Hz on one of the SC electrodes, whereas the differential conductance was recorded via a home-built current amplifier on the other one. DC bias was adjusted by the offset of the amplifier. We note that due to the geometry, the features of both QDs were measured simultaneously in a single measurement, and hence, the sum of two excitation spectra was captured. Out-of-plane magnetic field was realized by an AMI superconducting magnet.

### Modeling

In the theory, the QDs are modeled by capacitively interacting single sites (see Figure 1e) coupled to the SC with tunnel amplitudes *t*_T_ and *t*_B_. This restricts the electron number on the QDs between 0 and 2. The left SC in Figure 1e is handled in the zero bandwidth approximation^68,69^ effectively assuming quasi-particle sites with energy Δ_1_. The right superconducting probe with Δ_2_ superconducting gap is treated perturbatively with Dynes-like density of states. By allowing only a limited number of quasi-particles to be present in the system, the energy spectra with the eigenstates can be derived with exact diagonalization of the Fock-space Hamiltonians. Transition rates between the states, involving the processes of adding and removing an electron to the Andreev molecule, are expressed by Fermi’s golden rule. The transport (net current) is obtained by solving the classical master equation in the stationary limit, which governs the time evolution of the QD occupations (for more details see Supporting Information). Simulations with only capacitively interacting YSR states were also carried out. In those calculations, the QDs were coupled to two, separate single sites with energies Δ_1_ effectively turning off the superconducting hybridization between them. The simulated spectra are in good agreement with the sketches of Figure 3b,c (for further details see Supporting Information). In the model, we have used charging energies *U*_T_ = 1.2 meV and *U*_B_ = 2.2 meV, off-site repulsion energy (proportional to the interdot capacitance) *C* = 0.1 meV, and superconducting gaps of Δ_1_ = 200 μeV and Δ_2_ = 120 μeV, which were extracted from the measurements. Tunnel amplitudes were estimated as *t*_T_ = 0.15 meV and *t*_B_ = 0.05 meV based on the shape of the YSR states.

## Supporting Information Available

Detailed explanation of YSR states, further data analysis of both device A and B, and the description of the theoretical model (PDF)
