# Supplementary material for: Andreev Molecule in Parallel InAs Nanowires
Source: Nano Lett. 2021 Sep 20;21(19):7929–37. doi: 10.1021/acs.nanolett.1c01956 (PMC8517978; doi:10.1021/acs.nanolett.1c01956)
Supplement: Supplementary file 1 — nl1c01956_si_001.pdf [file nl1c01956_si_001.pdf]

Supplementary Notes for Andreev molecule in parallel InAs nanowires

Olivér Kürtössy,† Zoltán Scherübl,†,‡ Gergő Fülöp,† István Endre Lukács,¶ Thomas Kanne,§ Jesper Nygård,§ Péter Makk,*,† and Szabolcs Csonka*,†

†*Department of Physics, Budapest University of Technology and Economics and Nanoelectronics 'Momentum' Research Group of the Hungarian Academy of Sciences, Budafoki út 8, 1111 Budapest, Hungary*

‡*Univ. Grenoble Alpes, CEA, Grenoble INP, IRIG, PHELIQS, 38000 Grenoble, France*

¶*Center for Energy Research, Institute of Technical Physics and Material Science, Konkoly-Thege Miklós út 29-33., H-1121, Budapest, Hungary*

§*Center for Quantum Devices, Niels Bohr Institute,University of Copenhagen, 2100 Copenhagen, Denmark*

E-mail: makk.peter@ttk.bme.hu; csonka.szabolcs@ttk.bme.hu

YSR states in an intermediate limit

In superconducting QDs, sub-gap states can be formed by the hybridization of a QD level with the SC.[1–9] There are commonly used schemes to describe the formation of bound states: the two simplest of them is the superconducting atomic limit (or Andreev limit), where the superconducting gap is considered to be much larger than the charging energy of the QD ($\Delta \gg U$), and the opposite limit, the so-called Yu-Shiba-Rusinov limit ($\Delta \ll U$).

In the Andreev picture, quasi-particles are not allowed to appear in the SC. However, the superconductivity can couple the empty and doubly occupied QD states via Andreev reflection (AR) as shown in Fig. 1**a** leading to a singlet superposition of them. Depending on the coupling strength, the ground state is either the doublet (singly occupied QD) or the singlet, and transitions can be induced between them by changing the on-site potential exhibiting dispersively evolving excitation lines.

In the YSR limit, we first assume that the double occupation of the QD is forbidden as a result of the large U. Therefore the relevant number of electrons in the QD and quasi-particles in the SC is restricted to 0 or 1, as shown in Supp. Fig. 1**b**. In the following we use the notation of $|n, m\rangle$ for $|n\rangle_{\mathrm{QD}} \otimes |m\rangle_{\mathrm{SC}}$ and σ for a single spin. Due to the spin degeneracy, the $|0\rangle_{\mathrm{QD}}$, $|\uparrow\rangle_{\mathrm{QD}}$, $|\downarrow\rangle_{\mathrm{QD}}$ and $|0\rangle_{\mathrm{SC}}$, $|\uparrow\rangle_{\mathrm{SC}}$, $|\downarrow\rangle_{\mathrm{SC}}$ configurations in the QD and in the SC define 9 different states. When the total electron number parity of the system is even, the $|0, 0\rangle$ state can hybridize with the $|\uparrow, \downarrow\rangle - |\downarrow, \uparrow\rangle$ singlet, while in case of odd parity, the $|\sigma, 0\rangle$ and the $|0, \sigma\rangle$ doublets become coupled due to finite tunneling between the QD and the SC. Ground state transitions can be induced from the hybridized doublet to the hybridized singlet state (or from the singlet to the doublet) by changing the on-site potential, similarly to the Andreev picture.

Now we briefly discuss an intermediate limit, where both U and Δ are finite and have the same order of magnitude. Compared to the YSR limit, the double occupation of the QD is allowed, while the number of quasi-particles is still restricted to 0 or 1. In this system, the occupations of $|0\rangle_{\mathrm{QD}}$, $|\uparrow\rangle_{\mathrm{QD}}$, $|\downarrow\rangle_{\mathrm{QD}}$, $|\uparrow\downarrow\rangle_{\mathrm{QD}}$ and $|0\rangle_{\mathrm{SC}}$, $|\uparrow\rangle_{\mathrm{SC}}$, $|\downarrow\rangle_{\mathrm{SC}}$ span 12 different states

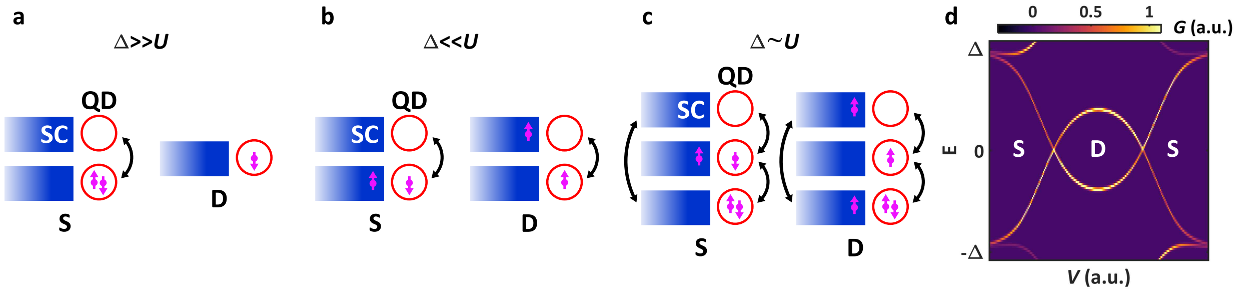

Supplementary Figure 1: **Formation of superconducting bound states. a-c** Hybridization processes in different regimes. The blue rectangles denote the SC electrode, whereas the red circle the QD. Different configurations if different rows are coupled by AR and tunneling processes (arrows). In the Andreev limit (**a**), the empty and double occupied QD states can hybridize via AR to form a singlet state (marked by "S"), while the doublet (marked by "D") remains intact. In the YSR limit (**b**), the double occupation of the QD is penalized. States with the same parity can hybridize in the presence of a finite tunnel coupling. In the intermediate limit (**c**), double occupation of the QD is allowed. Triplet states of parallel spins are excluded. **d** Simulated spectrum of a YSR state in the intermediate limit exhibiting an "eye-shaped" curve. The coupling strength was chosen as $t/U = 0.15$ and $\Delta/U = 0.2$ was used. Transport takes place when a ground state transition is induced.

altogether. Analogously to the YSR limit, the tunneling implies hybridization within the singlet and doublet subspaces, as depicted in Supp. Fig. 1**c**. The states $|0,0\rangle$, $|\uparrow,\downarrow\rangle - |\downarrow,\uparrow\rangle$, $|\uparrow\downarrow,0\rangle$ in the singlet sector and states $|\sigma,0\rangle$, $|0,\sigma\rangle$, $|\uparrow\downarrow,\sigma\rangle$ in the doublet sector couple (by tunneling or AR) providing level-repulsions in the energy diagram. In transport experiments, one can probe the excitation energies via finite-bias spectroscopy leading to an "eye-shaped" curve as a function of a plunger gate voltage, as outlined above. A simulation showing such a curve is depicted in Supp. Fig. 1**d**, where the singlet and doublet grounds states are addressed with "S" and "D", respectively. We note that all of the calculated spectra introduced in the main text and here were derived in the framework of this intermediate limit with the only difference of allowing 2 quasi-particles in the system.

Uncoupled YSR states (device A)

In the main text, we showed the SEM micrograph and the transport model of the sample exhibiting interacting YSR states, called device B. Here we provide the same for the uncoupled one, device A, in Supp. Fig. 2.

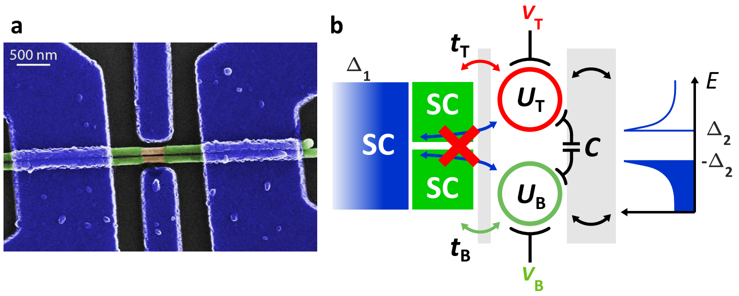

Supplementary Figure 2: **Device hosting the uncoupled YSR states.** **a** False-color SEM micrograph of device A. Compared to device B (see Fig. 1**a** in the main text), the nanowires originally merged by the epitaxial Al got disconnected and moved further away from each other. **b** Schematic illustration of device A, similarly to Fig. 1**d** in the main text. As the nanowire branches (green) are only linked by the common SC (blue), CAR is strongly suppressed by the long distance. Due to the larger spatial separation, the interdot capacitance is also weaker.

A small gap visible between the wires along the segment covered by epitaxial Al is the indication of the wires being fallen apart during the manipulation process, thus the epitaxial SC link between the wires is missing. On one hand, as the probability of CAR decays with increasing spatial separation of the conducting channels,[10] it is strongly suppressed in device A as it can take place between the QDs only via the ex-situ evaporated common SC. This results in an effective distance of ∼800 nm between the QDs. On the other hand, the interdot capacitance of the two InAs branches is also reduced.

The evolution of the YSR states parallel to the top QD resonances was discussed in the main text. Now we demonstrate the expected and measured spectra in device A parallel to the bottom QD resonances. The data is shown in Supp. Fig. 3.

Similarly to Fig. 2, panel **a** illustrates the conductance as a function of the top and bottom plunger gate voltages in the normal state. Here the spectra are examined parallel

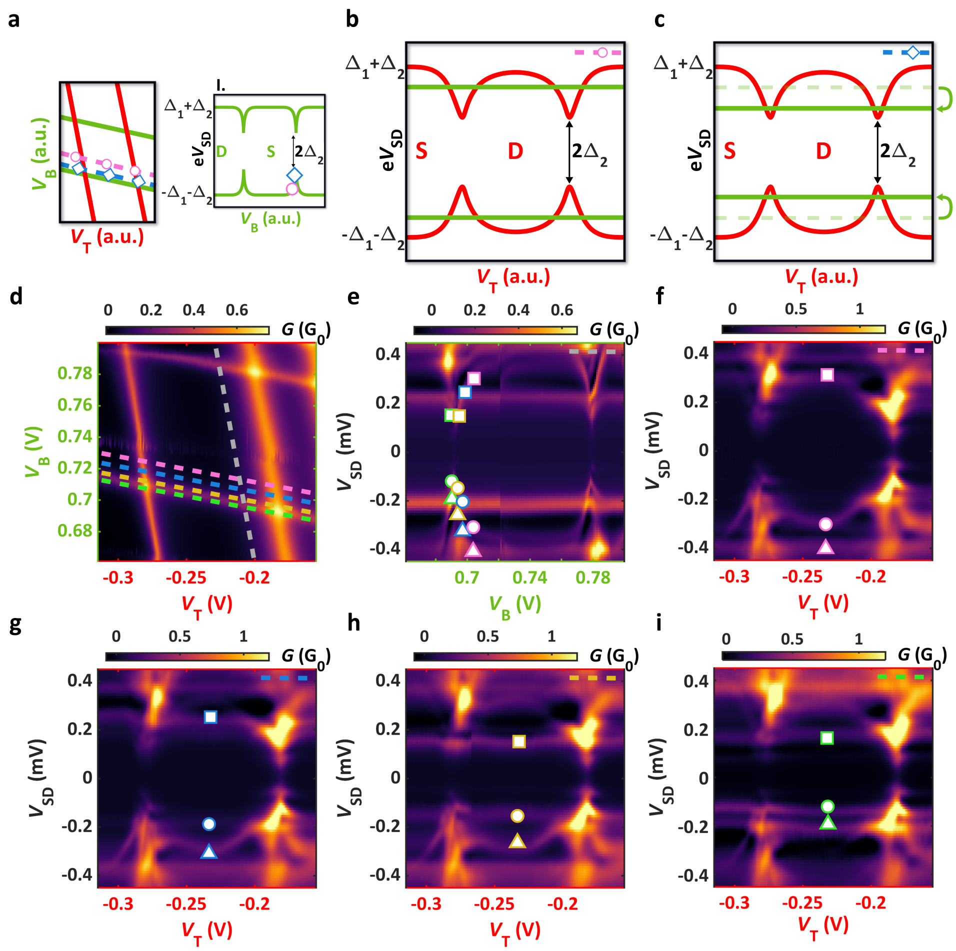

Supplementary Figure 3: **Additional data for device A**. **a** Sketch of the gate stability map as a function of V_T and V_B. The pink and blue lines indicate the line cuts along the spectra were studied. YSR_B spectrum with the markers upon is shown in inset **I.** to identify the corresponding energies along the cuts. **b** Predicted excitation spectrum along the pink line in panel **a**. YSR_T (red) is tuned by its own plunger gate and develops accordingly, while YSR_B (green) is kept on constant energy. **c** Similar spectrum as in panel **b**, but taken along the blue line, closer to the bottom QD resonance. The reduction of YSR_B excitation energy is expected (see green arrows) compared to the one in panel **b**. **d** Measured conductance as a function the plunger gates (same map as in Fig. 2**d**). **e** Measured excitation spectrum of the YSR_B states (same map as in Fig. 2**f**). The squares and circles indicate the dominant YSR_B, while the triangles indicate a more weakly coupled one. **f-i** Spectra along the lines shown in panel **d**. By moving closer to the bottom QD resonances, both YSR_B state energies are lowered providing double lines together with the unaffected YSR_T state. The low visibility of YSR_B comes from their low conductance compared to the "eye-shaped" YSR_T.

to the bottom QD resonances, therefore bias slices along the pink and blue cuts are taken. In panels **b** and **c**, the spectra along line cuts are presented off and close to resonance,

respectively, with the "eye-shaped" YSR_T (red) and the movement of YSR_B (green) depicted. We emphasize that YSR_B is weakly coupled, thus its excitation energy is expected to be at $\Delta_1 + \Delta_2$ except in the close vicinity of the resonances where it drops continuously to Δ_2. The weak coupling also implies the small conductance and the reduced visibility of YSR_B in the measurements compared to the much stronger features of YSR_T, which makes the tracking of its movements more difficult. Moreover, in the experiments, besides the dominant YSR_B highlighted in the main text and marked by square and circle symbols in panel **e**, the line of an additional YSR excitation can be observed. This state perhaps originates from another orbital of the bottom QD due to the finite level spacing and is marked by triangles in panel **e**. Nevertheless, the movements of both YSR_B states, which can be followed in panels **f-i**, are consistent with the green curves in panels **b** and **c**. The normal state gate stability map with dashed lines indicating where the spectra were taken is shown in panel **d**. When the line cuts are taken relatively far from the bottom QD resonance (panels **f-g**), the weakly coupled YSR_B marked with the triangles is barely visible and in energy is close to the gap edge. By approaching the resonances (panels **h-i**), both YSR_B excitations are lowered resulting in doubled constant energy lines crossing the "eye-shaped" YSR_T, whose conductance is sufficiently higher. All the individual symbols in panel **e** indicating the development of the YSR_B states can be assigned to their pairs in panels **f-i** consistently describing the movements.

Coupled YSR states (device B)

In the main text, a pair of bias slices were introduced from the measurements performed on device B revealing the hybridization of the YSR states (see Fig. 3**e-f**), which were compared to numerical simulations of the fully interacting system introduced. We present the spectra with the same gate settings with excluding the superconducting coupling and only allowing interdot Coulomb repulsion between the QDs. The results are depicted in Supp. Fig. 4.

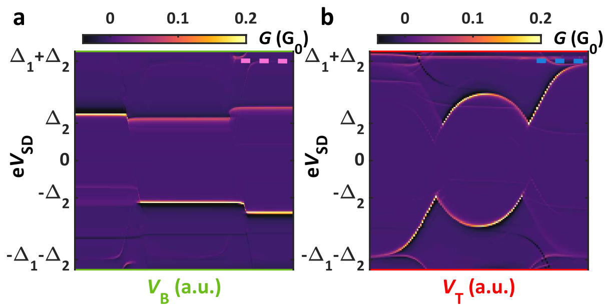

Supplementary Figure 4: **Simulations of the capacitively interacting YSR states. a** Numeric simulation using the parameter settings of Fig. 3**c**. The "step-like" shape of the top YSR state ($\mathrm{YSR_T}$) is reproduced. The bottom YSR state is suppressed, as its coupling was chosen three times smaller than the one for $\mathrm{YSR_T}$. b Similar simulation, but following the gate settings of Fig. 3**e**. The spectrum of the strongly coupled $\mathrm{YSR_T}$ is hardly disturbed by $\mathrm{YSR_B}$.

The simulations in panels **a** and **b** are derived along the pink and blue lines in Fig. 3**a** and 3**g** from the main text. As one can see, the spectra qualitatively match the sketches shown in Fig. 3**b-c**. Anti-crossings, dispersive lines, and distortions in the signals observed in the fully interacting case are absent. We note that the excitation lines of $\mathrm{YSR_B}$ are strongly suppressed due to the weak coupling of the bottom QD ($t_\mathrm{T} > t_\mathrm{B}$).

Here we provide additional spectra supported by numerical simulations along different traces in the gate stability map. The data is shown in Supp. Fig. 5.

In panel **a**, the honey-comb structure as a function of the plunger gate voltages is sketched. Here the pink line is parallel to the top QD resonance. Crossing the bottom QD resonances by increasing V_B results in an effective gating in the top QD, thus the pink line moves further away from the top QD resonance. The blue line set parallel to the bottom QD resonances follows similar behavior. Inset **I.** depicts $\mathrm{YSR_T}$ as a function of its own plunger gate, V_T with the pink square, circle, and diamond markers indicating the current energy along the pink line in the gate stability diagram. Analogously, inset **II.** depicts $\mathrm{YSR_B}$ as a function of V_B. As shown by the blue diamond, $\mathrm{YSR_B}$ is in the doublet ground state at small V_T. By increasing V_T the blue line crosses a triple point and $\mathrm{YSR_B}$ ends up in the singlet ground state as indicated by the blue circle and square. Panel **b** consists of the measured gate stability

map in the normal state in a larger window compared to the one in the main text. Panels **c** and **d** show large bias spectroscopy measurements accomplished in the normal state along the dotted lines in panel **b**. Coulomb diamonds were exhibited in a wide gate range in both nanowires and the charging energies were extracted as $U_{\mathrm{T}} = 1.2\,\mathrm{meV}$ and $U_{\mathrm{B}} = 2.2\,\mathrm{meV}$.

In panels **e** and **f** the expected and numerically simulated spectra of the capacitively interacting bound states along the pink cut from panel **a** are illustrated. Charging energies estimated from the measurements and tunnel amplitudes of $t_{\mathrm{T}} = 0.15\,\mathrm{meV}$ and $t_{\mathrm{B}} = 0.05\,\mathrm{meV}$ with $\Delta_1 = 200\ \mu\mathrm{eV}$ and $\Delta_2 = 120\ \mu\mathrm{eV}$ were used in the model. The off-site repulsion energy was set to $U_{\mathrm{C}} = 0.1\,\mathrm{meV}$. As the distance between the trace and the top QD resonance increases with V_{B} (due to effective gating of the Coulomb interaction) in panel **a**, $\mathrm{YSR_T}$ jumps from $\sim \Delta_2$ to higher energies when the bottom QD resonances are crossed (see the pink circle and square). Nonetheless, the spectrum changes drastically if the superconducting coupling between the QDs is involved (see panel **g**). Anti-crossings between $\mathrm{YSR_B}$ and $\mathrm{YSR_T}$ are induced (see white circles), and thus, the latter one bends to lower energy in the vicinity of the charge degeneracy points, similarly to Fig. 3**h** in the main text. The conductance of the weakly coupled $\mathrm{YSR_B}$ state (marked by the green arrows) is also enhanced in panel **g** compared to panel **f**. The corresponding experimental data (panel **h**) is in much better agreement with the spectrum derived from the fully interacting model. (i) Besides the anti-crossings being dominant (indicated by the white arrows), (ii) the conductance is greatly enhanced at $V_{\mathrm{B}} = 1.91\,\mathrm{V}$ predicted by the numerical simulation in panel **g**.

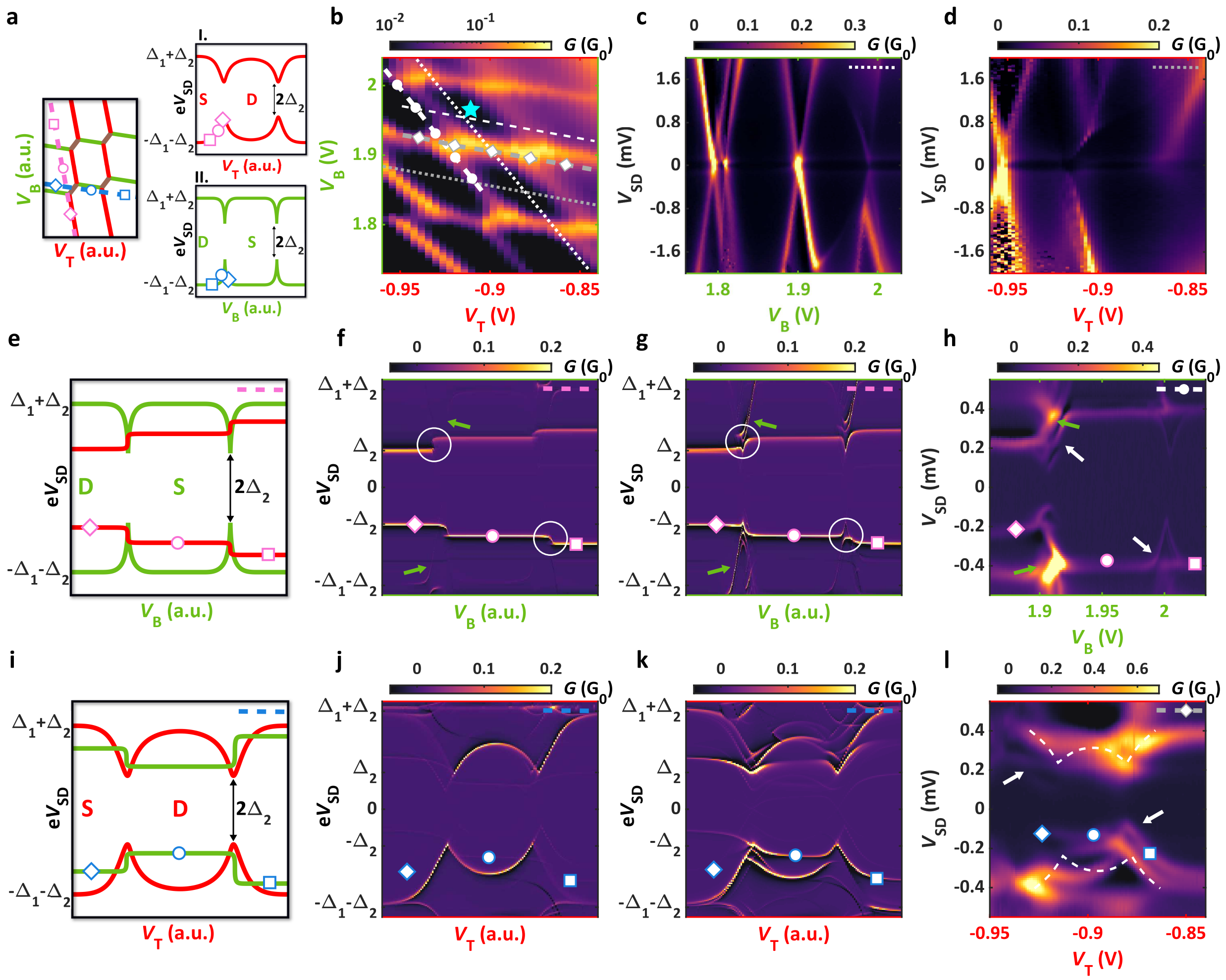

Supplementary Figure 5: **Supplementary data for the interacting YSR states (device B).** **a** Sketch of the gate stability map as a function of V_{T} and V_{B} in the presence of strong Coulomb interaction. Similarly to Fig. 3**a**, the pink and blue lines indicate the line cuts along the spectra were studied. Markers in the inset spectra guide identifying the bound state energies again. **b** Measured gate stability map in the normal state in a larger window compared to the one in the main text. **c-d** Large-bias spectroscopy of the QDs in the normal states along the dotted lines in panel **b**. **e-f** Predicted and simulated spectra along the pink line in panel **a** without and **g** with superconducting coupling introduced in the model. The superconducting coupling induces anti-crossings (white circles) and conductance enhancement (green arrows) as seen in the main text data. **h** Corresponding spectrum captured along the white dashed line in panel **b**. The highlighted anti-crossings (white arrows) and conductance enhancements (green arrows) are recovered in panel **g**, in the simulation of the fully interacting model. **i-k** Similar to panels **e-g**, but along the blue cut in panel **a**. Completely different development of the $\mathrm{YSR_B}$ signal is expected as the superconducting hybridization is turned on from the purely capacitively interacting case (panel **j**). **l** Finite-bias spectroscopy along the gray dashed line in panel **b**. The white dashed line shows the undisturbed $\mathrm{YSR_T}$ doublet measured far from the bottom QD resonances. All the key features, including the asymmetry in bias, doubling of the excitation lines and their dispersive evolution are in good agreement with the theory in panel **k**.

The hybridization is even more manifest in the slice parallel to the bottom QD resonances. Panel **i** in Supp. Fig. 5 illustrates the naive expectation of the capacitively interacting spectra along the blue cut in panel **a**. The numerical simulation in panel **j** qualitatively agrees with the sketch, nevertheless, we note that the shifts in the $\mathrm{YSR_B}$ excitation energies at the ground state transitions are smooth and continuous, and not abrupt. This phenomenon can be attributed to the number of electrons not being quantized in the singlet state since the ground state is the superposition of the empty and double occupied states in a standard YSR or Andreev picture. Panels **k** and **l** show the simulation of the fully interacting model and the relevant bias spectroscopy measurement along the gray dashed line in panel **b**, respectively. In resemblance to the data shown in the main text, (iii) bias asymmetry, (iv) distortions, and (v) the doubling of the excitation lines with their dispersive evolution (see white arrows in panel **k**) are observed matching the theory well in panel **j**.

We examined the excitation spectra far from any of the bottom QD resonances. Supp. Fig. 6**a** shows the gate stability map and the traces of the spectroscopy recorded (light blue star identifies the charge state in Supp. Fig. 5**b**). As one can see, the lines are selected parallel to the bottom QD resonance, however, they are captured deep in the blockade along the entire map. This resulted in the measured spectra given in panels **b** and **c**, where the "eye-shaped" $\mathrm{YSR_T}$ state is observed without any any signals of $\mathrm{YSR_B}$ or signatures of hybridization. These excitation lines of the undisturbed $\mathrm{YSR_T}$ doublet are indicated in Fig. 3**f** and Supp. Fig. 5**l** with the white dashed lines. It is also notable that the evolution of the $\mathrm{YSR_T}$ state is insensitive to the ground state of the $\mathrm{YSR_B}$ state as it is weakly coupled and evolves regardless of the parity of the electron number.

The deviation of the YSR states is the strongest when the excitation energies of both YSR states are similar. However, in these particular measurements, the bottom QD is in blockade, therefore $\mathrm{YSR_B}$ is bound to the gap edge with the energy of $\Delta_1 + \Delta_2$. Consequently, $\mathrm{YSR_B}$ is screened by the quasi-particle continuum, and interaction is suppressed and undetectable.[5] Therefore the visibility of the hybridization is especially restricted in the

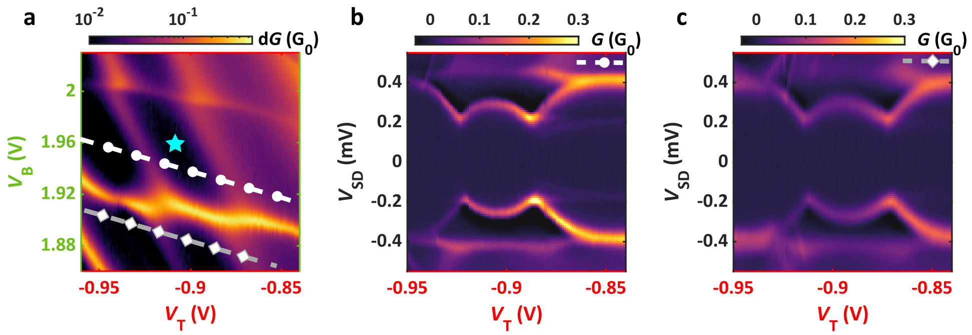

Supplementary Figure 6: **Undisturbed YSR state in device B. a** Gate stability map in the normal state (same as the one in Fig. 3 in the main text). The light blue star marks the charges state in 5**b**. **b** Bias spectroscopy measurement along the dashed line from panel **a**. The $\mathrm{YSR_T}$ state is unaffected by the $\mathrm{YSR_B}$ one, which is bound to gap edge at $\Delta_1 + \Delta_2$ energy. **c** Similar to panel **b**, but measured along the other cut from panel **a** where $\mathrm{YSR_B}$ occupies different ground state parity.

spectroscopy measurements accomplished along $\mathrm{YSR_T}$.

Andreev molecule in the Kondo regime

We also investigated the bound states in other gate ranges and we observed hybridization between the resonances of the two QDs. Here we report another interesting example when an Andreev molecule forms in the Kondo regime. Supp. Fig. 7 summarizes the measurements, which were carried out for more open QDs by applying higher gate voltages. Panel **a** shows the normal state gate stability map. The conductance of the top QD around T $= 1.2\,$V is particularly high, originating from Kondo physics. When the superconductivity is switched on it can still dominate the Kondo effect as the YSR states remain visible, thus suggesting $k_\mathrm{B}T_\mathrm{K} <= \Delta_1 = 200\ \mu\mathrm{eV}$.[3,11,12] Panels **b** and **c** show bias spectroscopy measurements along the white dashed lines in panel **a**. The line cuts are parallel to the top QD resonances, hence the bottom YSR states are expected to be tuned. While the development of a single YSR state can be followed with the white dashed line in panel **b**, it does not fit in panel **c** exhibiting a complex spectrum. Nevertheless, the bends of low-energy excitations belonging to the top QD ($V_\mathrm{B} = 0.92$ V) are still observable.

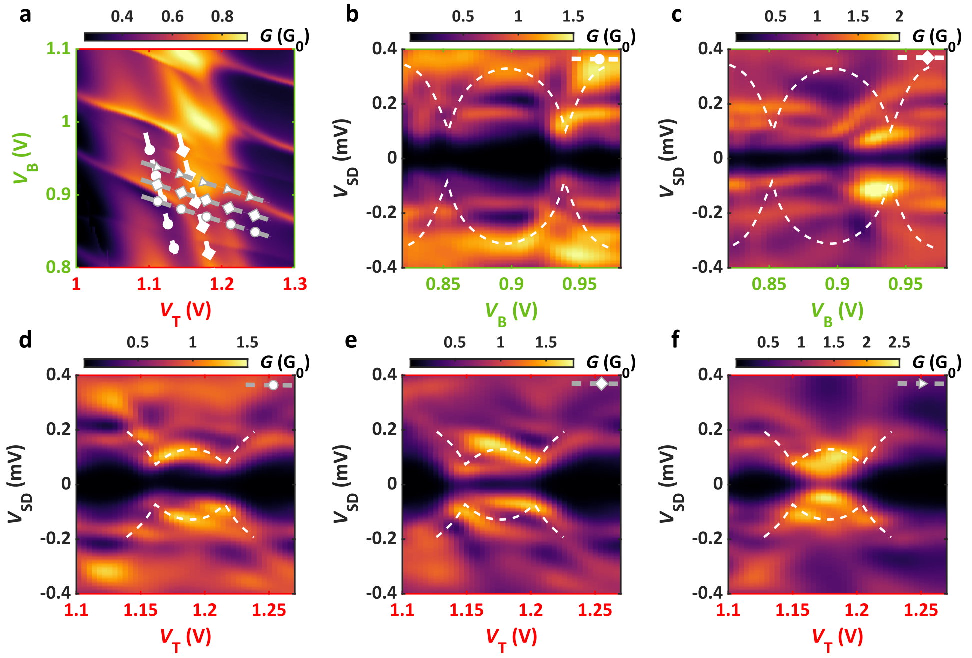

Supplementary Figure 7: **Interacting YSR states in a different gate range**. **a** Gate stability sweep in the normal state at more positive plunger gate voltages. The Kondo effect emerged in the top QD. **b-c** Bias spectroscopy measurements along the white lines in panel **a**. The bottom YSR (outlined in panel **b** with the dashed line) disappeared when the trace of the line cut entered the Kondo regime of the top QD. However, the bends towards zero energy in the top YSR signals were still observable. **d-f** Measured spectra along the gray lines in panel **a**. A usual YSR state is distinguishable from the rest in panel **d** (indicated the white dashed line), which is distorted as the bottom QD is brought to resonance and the conductance is enhanced.

Panels **d**, **e**, and **f** present the bias spectroscopy measurements along the gray lines in panel **a**. Now the slicing is parallel to the bottom QD resonances providing the evolution of the top YSR states. A dominant "eye-shaped" excitation appears in the rich spectrum (see the white dashed line in panel **d**), which changes completely as we approach the bottom QD resonances. While the excitation lines are doubled in panel **e** as seen earlier in Supp. Fig. 5**l**, the top YSR signal deviates from the usual shape and turns into a concave curve from convex one as a function of V_{T} in panel **f** suggesting the strong interaction of the two QDs. As further sub-gap states appeared in the spectra, the single YSR state picture can not be applied anymore to describe the system. Albeit the spectra became more complex due to the multiple excitation lines and their broadenings dropping the visibility of the hybridization

the signatures of the Andreev molecular state are still present.

Modeling

In this Supplementary Note we outline the framework used in the main text to simulate the transport spectrum of the Andreev molecule. First, the Hamiltonian is introduced, then we discuss the transport calculation.

The system consists of two, parallel-coupled quantum QDs and two superconducting electrodes as depicted in Fig. **1e**. One of the SC is strongly coupled to the QDs, their hybridization forms the YSR states. The other SC is weakly coupled.

The total Hamiltonian of the system is

$$H = H_{\mathrm{DQD}} + H_{\mathrm{SC1}} + H_{\mathrm{SC2}} + H_{\mathrm{T1}} + H_{\mathrm{T2}}. \tag{S1}$$

The first term describes the double QD,

$$H_{\mathrm{DQD}} = \sum_{\alpha=\mathrm{T,B}} \left(\varepsilon_\alpha n_\alpha + U_\alpha n_{\alpha\uparrow} n_{\alpha\downarrow}\right) + C n_{\mathrm{T}} n_{\mathrm{B}}, \tag{S2}$$

where $n_{\alpha\sigma} = d^\dagger_{\alpha\sigma} d_{\alpha\sigma}$ is the number of electrons with spin σ on QD_α, with $d^{(\dagger)}_{\alpha\sigma}$ being the annihilation (creation) operator of electrons with spin σ on QD_α and $\alpha = \mathrm{T,B}$ denotes the top, bottom QD. The parameters, ε_α and U_α are the level position and the on-site Coulomb energy of QD_α, and C is the interdot Coulomb repulsion.

The strongly coupled SC is described on the level of the zero bandwidth approximation (ZBA), i.e. it is considered as a single-site SC.[7,13–15] This approximation allows for the exact diagonalization of the $\mathrm{QD_T}$–SC1–$\mathrm{QD_B}$ subsystem's Hamiltonian to obtain the energy spectrum of the Andreev molecule. The ZBA superconductor Hamiltonian is

$$H_{\mathrm{SC1}} = \Delta_1 \left(c^\dagger_{\mathrm{SC1}\uparrow} c^\dagger_{\mathrm{SC1}\downarrow} + c_{\mathrm{SC1}\downarrow} c_{\mathrm{SC1}\uparrow}\right), \tag{S3}$$

where $c^{(\dagger)}_{\mathrm{SC1}\sigma}$ is the annihilation (creation) operator of an electron with spin σ in the SC and Δ_1 is the superconducting gap. This Hamiltonian can be diagonalized by a Bogoljubov-transformation, $c_\sigma = \frac{1}{\sqrt{2}}\left(\gamma_\sigma - \sigma\gamma^\dagger_{\bar{\sigma}}\right)$ obtaining

$$H_{\mathrm{SC1}} = \Delta_1 \sum_\sigma \gamma^\dagger_\sigma \gamma_\sigma. \tag{S4}$$

The tunnel coupling between superconductor SC1 and the QDs writes as

$$H_{\mathrm{T1}} = \sum_{\alpha=\mathrm{T,B}} t_\alpha \sum_\sigma \left(d^\dagger_{\alpha\sigma} c_{\mathrm{SC1}\sigma} + c^\dagger_{\mathrm{SC1}\sigma} d_{\alpha\sigma}\right), \tag{S5}$$

where t_α is tunneling amplitude. Using the Bogoljubov-transformation above this Hamiltonian translates to

$$H_{\mathrm{T1}} = \frac{1}{\sqrt{2}} \sum_{\alpha\sigma} t_\alpha \left[d^\dagger_{\alpha\sigma}\left(\gamma_\sigma - \sigma\gamma^\dagger_{\bar{\sigma}}\right) + \left(\gamma^\dagger_\sigma - \sigma\gamma_{\bar{\sigma}}\right) d_{\alpha\sigma}\right]. \tag{S6}$$

The already defined three Hamiltonian terms, $H_{\mathrm{DQD}} + H_{\mathrm{SC1}} + H_{\mathrm{T1}}$ are numerically diagonalized to obtain the energy spectrum and wavefunction of the Andreev molecular state.

In Eq. S1 H_{SC2} and H_{T2} describes the second superconducting lead and its weak tunnel coupling to the QDs, respectively. The superconductor SC2 is described by the BCS Hamiltonian,

$$H_{\mathrm{SC2}} = \sum_{\boldsymbol{k}\sigma} \varepsilon_{\mathrm{SC2}\boldsymbol{k}} c^\dagger_{\mathrm{SC2}\boldsymbol{k}\sigma} c_{\mathrm{SC2}\boldsymbol{k}\sigma} + \Delta_2 \sum_{\boldsymbol{k}} \left(c^\dagger_{\mathrm{SC2}\boldsymbol{k}\uparrow} c^\dagger_{\mathrm{SC2}-\boldsymbol{k}\downarrow} + c_{\mathrm{SC2}-\boldsymbol{k}\downarrow} c_{\mathrm{SC2}\boldsymbol{k}\uparrow}\right), \tag{S7}$$

where $c^{(\dagger)}_{\mathrm{SC2}\boldsymbol{k}\sigma}$ is the annihilation (creation) operator for electrons with momentum $\boldsymbol{k}$ and spin σ in the SC2 superconductor, $\varepsilon_{\mathrm{SC2}\boldsymbol{k}}$ is normal state dispersion and Δ_2 is superconducting gap. Note that here both superconducting gaps, Δ_1 and Δ_2 are assumed to be real, the possible effects originating from the superconducting phase difference are neglected. The

tunnel coupling Hamiltonian is

$$H_{\mathrm{T2}} = t_{\mathrm{SC2}} \sum_{\alpha \boldsymbol{k} \sigma} \left(d^{\dagger}_{\alpha\sigma} c_{\mathrm{SC2}\boldsymbol{k}\sigma} + c^{\dagger}_{\mathrm{SC2}\boldsymbol{k}\sigma} d_{\alpha\sigma} \right), \tag{S8}$$

where t_{SC2} is tunneling amplitude, for the simplicity, assumed to be the same for the two QDs.

The tunnel coupling to SC2 is assumed to be weak and treated perturbatively using Fermi's golden rule. In this description the tunnel coupling induces transitions between eigenstates of the $\mathrm{QD_T}$–SC1–$\mathrm{QD_B}$ system. The time evolution of the occupation of the eigenstates $|\chi\rangle$, P_χ is governed by a master equation together with the normalization condition $\sum_\chi P_\chi = 1$,

$$\frac{dP_\chi}{dt} = \sum_{\chi' \neq \chi} \left(W_{\chi\chi'} P_{\chi'} - W_{\chi'\chi} P_\chi \right). \tag{S9}$$

Here $W_{\chi\chi'}$ the total transition rate from $|\chi'\rangle$ state to $|\chi\rangle$ induced by the tunnel coupling to SC2. The rates are the sum of two processes, when an electron is added to the Andreev molecule and when one is removed, i.e. $W_{\chi\chi'} = W_{\chi'\chi}\left(d^{\dagger}_{\alpha\sigma}\right) + W_{\chi'\chi}\left(d_{\alpha\sigma}\right)$. These two contributions are expressed as

$$\begin{aligned} W_{\chi'\chi}\left(d^{\dagger}_{\alpha\sigma}\right) &= \pi t^2_{\mathrm{SC2}} \left|\langle\chi'|d^{\dagger}_{\alpha\sigma}|\chi\rangle\right|^2 \rho_S\left(E_\chi - E_{\chi'} - \mu_{\mathrm{SC2}}\right) f\left(E_\chi - E_{\chi'} - \mu_{\mathrm{SC2}}\right) \\ W_{\chi'\chi}\left(d_{\alpha\sigma}\right) &= \pi t^2_{\mathrm{SC2}} \left|\langle\chi'|d_{\alpha\sigma}|\chi\rangle\right|^2 \rho_S\left(E_\chi - E_{\chi'} + \mu_{\mathrm{SC2}}\right) f\left(E_\chi - E_{\chi'} + \mu_{\mathrm{SC2}}\right), \end{aligned} \tag{S10}$$

where $\rho_S(E) = \rho_0 \mathrm{Re}\frac{E+i\gamma}{\sqrt{(E+i\gamma)^2 - \Delta_2^2}}$ is the Dynes-like density of states (DOS) in SC2 superconductor, with γ being the Dynes-parameter and ρ_0 is the normal state DOS, assumed to be constant, $f(E)$ is the Fermi function, E_χ denote the energy of the $|\chi\rangle$ state and $\mu_{\mathrm{SC2}} = eV_{\mathrm{SD}}$ is chemical potential difference of the two superconducting leads, due to the applied bias voltage, V_{SD}.

To derive the current through the device the master equation, Eq. S9 is solved in the

stationary limit, $dP_\chi/dt = 0$ to obtain the occupations. The current writes as

$$I = \frac{e}{\hbar} \sum_{\alpha\chi\chi'\sigma} \left[W_{\chi'\chi}\left(d_{\alpha\sigma}\right) - W_{\chi'\chi}\left(d^\dagger_{\alpha\sigma}\right) \right] P_\chi. \tag{S11}$$

The differential conductance is obtained as the derivative of the current, i.e. $G = e\frac{dI}{d\mu_{\mathrm{SC2}}}$.

For the case, when only capacitive coupling is assumed between the QDs, one has to eliminate the SC mediated tunneling processes that couples the states of the two QDs. An example for such a process is when a Cooper pair from the SC1 electrode splits between the QDs. As the $\mathrm{QD_T}$–SC1–$\mathrm{QD_B}$ subsystem is treated up to all orders in the tunneling, such processes are necessarily present in the description above. One can formally remove them by coupling the QDs to two separate SCs. This can be formulated in the following Hamiltonians:

$$H_{\mathrm{SC1}} = \Delta_1 \sum_{\alpha=\mathrm{T,B}} \left(c^\dagger_{\mathrm{SC1}\alpha\uparrow} c^\dagger_{\mathrm{SC1}\alpha\downarrow} + c_{\mathrm{SC1}\alpha\downarrow} c_{\mathrm{SC1}\alpha\uparrow} \right), \tag{S12}$$

$$H_{\mathrm{T1}} = \sum_{\alpha=u,l} t_{\mathrm{SC1}\alpha} \sum_\sigma \left(d^\dagger_{\alpha\sigma} c_{SC1\alpha\sigma} + c^\dagger_{SC1\alpha\sigma} d_{\alpha\sigma} \right). \tag{S13}$$

The difference compared to Eqs. S3&S5 is that the SC1 superconductor is split into two parts, which are only coupled to one of the QDs, This way the tunnel coupling hybridize the QD and SC states to local YSR states, but the further hybridization of the YSR state into molecular states are prevented. The rest of the transport calculation is the same as above.

References

(1) Buitelaar, M.; Nussbaumer, T.; Schönenberger, C. Quantum dot in the Kondo regime coupled to superconductors. *Physical Review Letters* **2002**, *89*, 256801.

(2) Sand-Jespersen, T.; Paaske, J.; Andersen, B. M.; Grove-Rasmussen, K.; Jørgensen, H. I.; Aagesen, M.; Sørensen, C.; Lindelof, P. E.; Flensberg, K.; Nygård, J.

Kondo-enhanced Andreev tunneling in InAs nanowire quantum dots. *Physical review letters* **2007**, *99*, 126603.

(3) Eichler, A.; Weiss, M.; Oberholzer, S.; Schönenberger, C.; Yeyati, A. L.; Cuevas, J.; Martín-Rodero, A. Even-odd effect in Andreev transport through a carbon nanotube quantum dot. *Physical Review Letters* **2007**, *99*, 126602.

(4) Grove-Rasmussen, K.; Jørgensen, H. I.; Andersen, B. M.; Paaske, J.; Jespersen, T. S.; Nygård, J.; Flensberg, K.; Lindelof, P. E. Superconductivity-enhanced bias spectroscopy in carbon nanotube quantum dots. *Physical Review B* **2009**, *79*, 134518.

(5) Pillet, J.; Quay, C.; Morfin, P.; Bena, C.; Yeyati, A. L.; Joyez, P. Andreev bound states in supercurrent-carrying carbon nanotubes revealed. *Nature Physics* **2010**, *6*, 965–969.

(6) Lee, E. J.; Jiang, X.; Houzet, M.; Aguado, R.; Lieber, C. M.; De Franceschi, S. Spin-resolved Andreev levels and parity crossings in hybrid superconductor–semiconductor nanostructures. *Nature nanotechnology* **2014**, *9*, 79–84.

(7) Jellinggaard, A.; Grove-Rasmussen, K.; Madsen, M. H.; Nygård, J. Tuning Yu-Shiba-Rusinov states in a quantum dot. *Phys. Rev. B* **2016**, *94*, 064520.

(8) Scherübl, Z.; Fülöp, G.; Moca, C. P.; Gramich, J.; Baumgartner, A.; Makk, P.; Elalaily, T.; Schönenberger, C.; Nygård, J.; Zaránd, G.; Csonka, S. Large spatial extension of the zero-energy Yu–Shiba–Rusinov state in a magnetic field. *Nature communications* **2020**, *11*, 1–9.

(9) Prada, E.; San-Jose, P.; de Moor, M. W.; Geresdi, A.; Lee, E. J.; Klinovaja, J.; Loss, D.; Nygård, J.; Aguado, R.; Kouwenhoven, L. P. From Andreev to Majorana bound states in hybrid superconductor–semiconductor nanowires. *Nature Reviews Physics* **2020**, *2*, 575–594.

(10) Recher, P.; Sukhorukov, E. V.; Loss, D. Andreev tunneling, Coulomb blockade, and resonant transport of nonlocal spin-entangled electrons. *Physical Review B* **2001**, *63*, 165314.

(11) Van der Wiel, W.; De Franceschi, S.; Fujisawa, T.; Elzerman, J.; Tarucha, S.; Kouwenhoven, L. The Kondo effect in the unitary limit. *Science* **2000**, *289*, 2105–2108.

(12) Eichler, A.; Deblock, R.; Weiss, M.; Karrasch, C.; Meden, V.; Schönenberger, C.; Bouchiat, H. Tuning the Josephson current in carbon nanotubes with the Kondo effect. *Physical Review B* **2009**, *79*, 161407.

(13) Affleck, I.; Caux, J.-S.; Zagoskin, A. M. Andreev scattering and Josephson current in a one-dimensional electron liquid. *Phys. Rev. B* **2000**, *62*, 1433–1445.

(14) Probst, B.; Domínguez, F.; Schroer, A.; Yeyati, A. L.; Recher, P. Signatures of nonlocal Cooper-pair transport and of a singlet-triplet transition in the critical current of a double-quantum-dot Josephson junction. *Phys. Rev. B* **2016**, *94*, 155445.

(15) Grove-Rasmussen, K.; Steffensen, G.; Jellinggaard, A.; Madsen, M. H.; Zitko, R.; Paaske, J.; Nygård, J. Yu-Shiba-Rusinov screening of spins in double quantum dots. *Nature Communications* **2018**, *9*, 2376.
